# Evaluation of the antibacterial and anticancer activities of some South African medicinal plants

**DOI:** 10.1186/1472-6882-11-14

**Published:** 2011-02-17

**Authors:** Mary A Bisi-Johnson, Chikwelu L Obi, Toshio Hattori, Yoshiteru Oshima, Shenwei Li, Learnmore Kambizi, Jacobus N Eloff, Sandeep D Vasaikar

**Affiliations:** 1 Department of Medical Microbiology, Walter Sisulu University, Mthatha 5117, South Africa; 2 Division of Academic Affairs & Research, Walter Sisulu University, Mthatha 5117, South Africa; 3 Department of Emerging Infectious Diseases, School of Medicine, Postgraduate Division, Tohoku University, Sendai, Japan; 4 Department of Natural Products Chemistry, Graduate School of Pharmaceutical Sciences, Tohoku University, Sendai, Japan; 5 Department of Botany, Walter Sisulu University, Mthatha 5117, South Africa; 6 Phytomedicine Programme, Department of Paraclinical Science, Faculty of Veterinary Medicine, University of Pretoria, Onderstepoort 0110, South Africa

## Abstract

**Background:**

Several herbs are traditionally used in the treatment of a variety of ailments particularly in the rural areas of South Africa where herbal medicine is mainly the source of health care system. Many of these herbs have not been assessed for safety or toxicity to tissue or organs of the mammalian recipients.

**Methods:**

This study evaluated the cytotoxicity of some medicinal plants used, inter alia, in the treatment of diarrhoea, and stomach disorders. Six selected medicinal plants were assessed for their antibacterial activities against ampicillin-resistant and kanamycin-resistant strains of *Escherichia coli *by the broth micro-dilution methods. The cytotoxicities of methanol extracts and fractions of the six selected plants were determined using a modified tetrazolium-based colorimetric assay (3-(4, 5-dimethylthiazol)-2, 5-diphenyl tetrazolium bromide (MTT) assay).

**Results:**

The average minimum inhibitory concentration (MIC) values of the plants extracts ranged from 0.027 mg/mℓ to 2.5 mg/mℓ after 24 h of incubation. *Eucomis autumnalis *and *Cyathula uncinulata *had the most significant biological activity with the least MIC values. The in vitro cytotoxicity assay on human hepatocarcinoma cell line (Huh-7) revealed that the methanol extract of *E. autumnalis *had the strongest cytotoxicity with IC_50 _of 7.8 μg/mℓ. Ethyl acetate and butanol fractions of *C. uncinulata, Hypoxis latifolia, E. autumnalis *and *Lantana camara *had lower cytotoxic effects on the cancer cell lines tested with IC_50 _values ranging from 24.8 to 44.1 μg/mℓ; while all the fractions of *Aloe arborescens *and *A. striatula *had insignificant or no cytotoxic effects after 72 h of treatment.

**Conclusions:**

Our results indicate that the methanol fraction of *E. autumnalis *had a profound cytotoxic effect even though it possessed very significant antibacterial activity. This puts a query on its safety and hence a call for caution in its usage, thus a product being natural is not tantamount to being entirely safe. However, the antibacterial activities and non-cytotoxic effects of *A. arborescens *and *A. striatula *validates their continuous usage in ethnomedicine.

## Background

Various plants are used in the treatment of gastrointestinal related diseases. Several studies have documented reports on some herbs used in ethnotherapy of diarrhea, dysentery, vomiting, stomach cramps and other associated ailments [1-3]. Contrary to the belief of a large proportion of the populace that anything natural is safe, many commonly used herbs cause acute toxicity effects and in the long term may be toxic. The toxic effects may range from diarrhoea, hypersensitivity reactions, nausea or vomiting, to organ-targeted toxicity; immunotoxicity, embryo/foetal and prenatal toxicity, mutagenicity/genotoxicity, hepatotoxicity, nephrotoxicity, presence of epileptogenic compounds, cardiac toxins, gastrointestinal toxins to carcinogenicity [4]. Other adverse effects of herbal medicines may include cardiovascular, neurological and dermatologic toxic effects. In the review by Luyckx and Naicker [5], it was stated that 'drug-induced nephrotoxicity reportedly contributes to up to 26% of cases of hospital-acquired acute kidney injury (AKI) and 18% of cases of global community-acquired AKI ...' The review [5] further revealed that folk remedies account for up to 35% of cases of AKI in the developing world.

Many of the plants widely acclaimed to be of therapeutic values have not enjoyed vigorous assessments to gauge their safety. A number of cases of complications arising after the administration of medicinal herbs have been reported. Foyaca-Sibat and co-investigators described the case reports of two patients with neuromyotonia not associated with malignancies. The patients were reported to have developed acute renal failure while under treatment with herbal medications by their traditional healer in the former Transkei region of South Africa [6].

Other researchers have also identified medicinal plants with potential toxicity such as the extracts of Athrixia phylicoides DC. (Bush tea) [7]; and a flavonol glycoside from *Bauhinia galpinii *[8]. Genotoxicity and mutagenic effects in the *Salmonella *microsome assay have been reported in *Crinum macowanii*, *Chaetacme aristata *Planch. (Celastraceae), *Plumbago auriculata *Lam. (Plumbaginaceae), *Catharanthus roseus *(L.) G.Don. (Apocynaceae) and *Ziziphus mucronata *Willd. (Rhamnaceae) [9]. Additionally, Michellamine B-an alkaloid dimmers isolated from *Ancistrocladus korupensis *was inhibitory to several laboratory and clinical strains of HIV-1, including the AZT resistant strain G910-6 and the pyridinone-resistant strain A17; as well as strains of HIV-2. However, the high toxicity of this compound to several human cell lines prevented its further evaluation [10]. Data on the cytotoxic assessments of herbs are very few compared with the huge number of plants acclaimed to have therapeutic values [11,12]. This study investigated the cytotoxic effects of *Aloe arborescens, A. striatula, Cyathula uncinulata, Eucomis autumnalis, Hypoxis latifolia *and *Lantana camara *commonly used in the treatment of gastrointestinal infections in the Oliver R. Tambo District Municipality (ORTDM), Eastern Cape Province, South Africa.

## Methods

### Plant material, extraction and fractionation

Fresh plant parts were collected in ORTDM, Eastern Cape Province, South Africa between July 2008 and February 2010. The plants were identified in the Kei herbarium, Walter Sisulu University, South Africa where voucher specimens have been deposited. Information on the selected herbs are presented in Table 1[13-19]. The air-dried plant parts were extracted three times with methanol (Merck, Japan) and filtered using a Buchner funnel and Whatman No. 1 filter paper. The extracts were concentrated under reduced pressure at a temperature of 40°C using a rotating evaporator to yield methanol extract. The methanol extract was then suspended in deionised water and partitioned sequentially with Ethyl acetate and n-butanol. The fractions were concentrated under reduced pressure to yield the corresponding fractions and the remaining water fraction.

**Table 1:** Selected plants investigated and their usage

HERB	LOCAL NAME	PLANTPART	USES AND REFERENCE
*Aloe arborescens *Mill ASHODELACEAE	Ikhala Inkalane encane (Z)	Leaves	Leaf decoction for diarrhoea [13], effective burn treatment [14]

*Aloe striatula* ASHODELACEAE	Inkalana (X) Intelezi (Ng)	Leaves	Leaf decoction for diarrhoea [13], purgative, expels worms

*Cyathula uncinulata* AMARANTHACEAE	Isinama (X)	Leaves	Leaf decoction for HIV treatment [13], stomach ailment

*Eucomis autumnalis* HYACINTHACEAE	Ubhulungu becanti (X) Umathunga (Z)	Root	Decoctions of bulb and roots for coli, flatulence [15].

*Hypoxis latifolia* HYPOXIDACEAE	Ilabatheka (X, Z)	Root	Treat benign prostrate [16]; Headaches, dizziness, mental disorders, HIV inflammation [17]; Boil ground dried tuber for diarrhoea.

*Lantana camara* VERBENACEAE	Ndzindzibila (X) Ubuhobe besikhiwa (N) Mbarapati (S)	Leaves Flower	Leaf decoction for boosting immune system in HIV patients [Nomvula Twaise, personal communication], antivirus [18], treatment of wound [19].

N - Ndebele; Ng - Nguni; S - Shona; × - Xhosa; Z - Zulu

### Antibacterial assay

The antibacterial assay was by the determination of minimum inhibitory concentration values of plant extracts and fractions against Gram negative bacteria strains. The broth dilution method was carried out in 96-well microtitre plates using ampicillin-resistant and kanamycin-resistant strains of *Escherichia coli *as the test organisms. A McFarland No1 standard suspension of bacteria inoculum was prepared in sterile Mueller Hinton Broth. Triplicate tests were performed in a series of two-fold dilutions of extract (10 mg/mℓ) as previously described [20]. Kanamycin was used as the positive control for ampicillin-resistant *E. coli s*train while ampicillin was used in the case of kanamycin-resistant *E. coli *strain. Plates were incubated at 37°C for 18 h and an hour before the end of incubation, 40 μℓ of 0.2 mg/ml INT (*p*-iodonitrotetrazolium salt) solution was added to each well. The lowest concentration indicating inhibition of growth was recorded as the MIC. This was indicated by the clear well after further incubation with INT as opposed to the pinkish colouration in growth wells.

### Cytotoxicity assays

The in vitro cytotoxicities of the selected herbs and solvent-solvent fractions on a human hepatoma cell line, (Huh-7), which was established from a hepatocellular carcinoma were examined using a modified MTT assay [21]. The Huh-7 was maintained at - 80°C in Dulbecco's Modified Eagle Medium (DMEM) and recovered from preservative by centrifugation. The pellet was re-suspended in fresh DMEM and cultured in a humidified atmosphere at 37°C using RPMI 1640 supplemented with 10% foetal bovine serum, 100 U/mℓ penicillin G and 100 ug/ml streptomycin and L-glutamine (Gibco BRL) in 5% CO_2 _incubator (Thermo Fischer Scientific, Wakenyaku Co. Ltd, Japan). Cells were sub-cultured every 2 days after confluent growth was observed.

The MTT assay was carried out as follows. Briefly, the cells at a density of 1 × 10^4 ^per mℓ were seeded in each well of a flat-bottom 96-well plate containing 100 μℓ of the growth medium. Cells were permitted to adhere for 24 h, and then treated with various fractions at concentrations 0, 1, 10 and 100 μg/mℓ for 72 h. After that, 20 μℓ of 5 mg/mℓ MTT in phosphate buffered saline (PBS) was added to each well and the plate was incubated for an additional 2 h. The medium was discarded and the formazan blue, which formed in the cells, was dissolved with 100 μℓ MTT stop solution (Triton-X100-20 mℓ; Isopropyl alcohol-500 mℓ; HCl-2 mℓ). After incubation at 37°C for 10 min, the absorbance of the dissolved solutions was detected at 490 nm on a microplate ELISA reader (Thermo Labsystems, Japan). Cytotoxicity was expressed as the concentration of extracts or fractions inhibiting cell growth by 50% (IC_50_). All tests and analyses were run in triplicate. Statistical analyses were carried out using MS Excel 2007.

## Results

### Antibacterial activities

**Figure 1:**
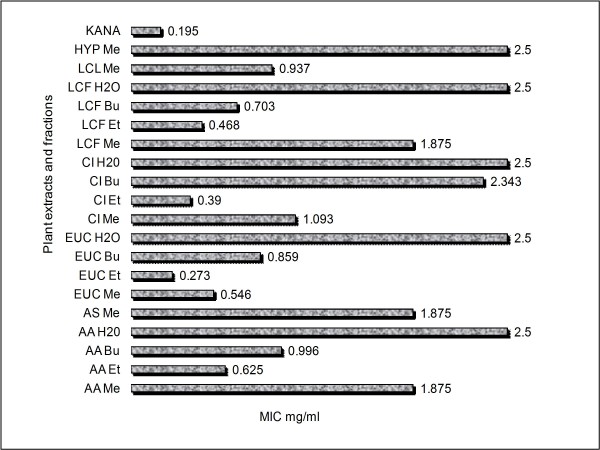
**Minimum inhibitory concentration of plant extracts and fractions**. AA Me = Methanol fraction of *A. arborescens (AA)*; AA Et = Ethyl acetate fraction of AA; AA Bu = n-Butanol fraction of *AA*; AA H2O = Water fraction of AA; AS Me = Methanol fraction of *A. striata*; EUC Me = Methanol fraction of *E. autumnalis *(EA); EUC Et = Ethyl acetate fraction of *EA*; EUC Bu = n-Butanol fraction of *EA*; EUC H2O = Water fraction of *EA*; CI Me = Methanol fraction of *C. uncinulata *(CU); Et = Ethyl acetate fraction of *CU*; CI Bu = n-Butanol fraction of *CU*; CI H2O = Water fraction of *CU*; LCF Me = Methanol fraction of *L. camara *fruit/flower *(LCF)*; LCF Et = Ethyl acetate fraction of *LCF *; LCF Bu = n-Butanol fraction of *LCF*; LCF H2O = LCF Water fraction of *LCF*; LCL Me = Methanol fraction of *L. camara *leaves; HYP Me = HYP Methanol fraction.

Active antibacterial extracts were revealed as clear spots of inhibition of the growth of test organisms in microtitre wells. The pinkish contrasting wells of bacterial growth was indicative of non-cleavage of the tetrazolium salt to yield the pinkish or purplish formazan products [22]. The average MIC values of the plants extracts ranged from 0.27 mg/mℓ and 2.5 mg/mℓ after 24 h of incubation (Figure 1). The Ethyl acetate fraction of each type of plant was the most active fraction against the test bacteria. *E. autumnalis *had the least MIC (0.27 mg/mℓ) followed by *C. uncinulata *(0.39 mg/mℓ) and thus demonstrated good antibacterial activities. The methanol extract of *H. latifolia *had the highest value of MIC (2.5 mg/mℓ).

### Cytotoxicities of methanol extract of plants extract and fractions

The results of cytotoxicity assays of the extracts and fractions of the investigated plants on human hepatoma cell line (Huh-7) are shown in Table 2. The methanol extract of *E. autumnalis *(IC_50 _7.8 μg/mℓ) was more cytotoxic than the control toxic substance, berberine (IC_50 _9.8 μg/mℓ), other plant extracts and solvent fractions. Of the solvent fractions, the Ethyl acetate and butanol fractions of *C. uncinulata, H. latifolia, E. autumnalis *and *L. camara *had moderate cytotoxic effects on the cancer cell lines tested with IC_50 _values ranging from 24.8 to 44.1 μg/mℓ while all the fractions of *A. arborescens *and *A. striatula *had insignificant or no cytotoxic effects after 72 h of treatment (IC_50 _1000 to >1000 μg/mℓ).

**Table 2:** Inhibitory concentration, IC_50 _(ug/ml) of selected herbs

CODE S/NO	SAMPLE CODE	EXTRACT FRACTION	IC_50 _(ug/ml)
1	AA Me	AA Methanol fraction	> 1000

2	AA Et	AA Ethyl acetate fraction	> 1000

3	AA Bu	AA n-Butanol fraction	> 1000

4	AA H2O	AA Water fraction	> 1000

5	AS Me	AS Methanol fraction	1000

6	EUC Me	EA Methanol fraction	7.8

7	EUC Et	EA Ethyl acetate fraction	28.5

8	EUC Bu	EA n-Butanol fraction	39.3

9	EUC H2O	EA Water fraction	379.0

10	CI Me	CI Methanol fraction	24.8

11	CI Et	CI Ethyl acetate fraction	36.3

12	CI Bu	CI n-Butanol fraction	30.0

13	CI H2O	CI Water fraction	714.0

14	LCF Me	LCF Methanol fraction	169.0

15	LCF Et	LCF Ethyl acetate fraction	44.1

16	LCF Bu	LCF n-Butanol fraction	150.0

17	LCF H2O	LCF Water fraction	1000

18	LCL Me	LCL Methanol fraction	161.0

19	HYP Me	HYP Methanol fraction	24.4

PC	Positive control	Berberine	9.8

AA - *Aloe arborescens; *AS - *Aloe striatula*; EA - *Eucomis autumnalis*; CI - *Cyathula uncinulata*; LCF - *Lantana camara (*flower/fruits); LCL - *Lantana camara *(leaves); HYP - *Hypoxis latifolia*.

## Discussion

This study evaluated the cytotoxicity and antibacterial activities of methanol extracts and solvent fractions of *A. arborescens, A. striatula, C. uncinulata, E. autumnalis, H. latifolia *and *L. camara*. Among all the samples, the methanol extract of *E. autumnalis *exhibited greatest cytotoxicity on the cell line tested. However, the water fraction of *E. autumnalis *and that of other plants showed insignificant cytotoxicity on the cell line, compared with the polar solvent fractions (Table 2).

The Ethyl acetate fraction of *E. autumnalis *had the least MIC (0.27 mg/mℓ) followed by *C. uncinulata *(0.39 mg/mℓ) against the test bacteria and thus demonstrated good antibacterial activities. Various biological activities of *Eucomis *were reported [23,24]. *E. autumnalis *is known for its anti-inflammatory and antispasmodic activities and these have been attributed to components such as homoisoflavones and flavonoids [25]. *E. autumnalis *also contains some steroidal triterpenoids which are known to be beneficial in wound therapy [25]. However, the bulb was reported to be toxic [25] agreeing very much with our findings.

Eucosterol glycoside, a lanosterol oligosaccharides isolated from *E. bicolor *demonstrated antitumor activity by causing 44% inhibition of TPA-stimulated ^32^P incorporation into phospholipids of HeLa. This activity has been suggested to probably relate to the use of bulb decoctions of *E. autumnalis *to relieve abdominal distensions and abdominal pain by the Tswana and Pedi tribes of South Africa [26]. According to Koorbanally *et al. *[27], sheep drenched with fresh bulbs of *E. autumnalis *in an animal feeding trial presented with listlessness, anorexia, foaming at the mouth, tympanites, an inactive rumen and a strong pulse leading eventually to death within twenty-four hours. Despite these submissions *Eucomis *is one of the most traded genera of plants in South Africa [28], hence the need for caution in their usage.

## Conclusions

In conclusion, the results obtained indicated that the methanol extract of *E. autumnalis *exhibited much greater cytotoxicity than the methanol extract and solvent fractions of all other plants investigated even though it had strong antibacterial activities. *E. autumnalis *showed selective anticancer activity against the human hepatoma cell line, whereas the two *Aloe *spp. were non toxic on the cell line. In addition, the study showed that *Aloe arborescens, A. striatula *and *C. uncinulata *may be candidate plants for eventual drug design. Medicinal plants are natural products and may have therapeutic potentials; however, being natural does not make them automatically safe.

## Competing interests

The authors declare that they have no competing interests.

## Authors' contributions

MAB participated in the design of the study, carried out field work, prepared the extracts, participated in the MTT assay and drafted the manuscript. CLO conceived of the study, participated in the design and coordination of the study, supervised the study and revised the manuscript. TH coordinated bench work between collaborators in South Africa and Japan and helped to revise the manuscript. YO was involved in coordination of study done in Japan. SL was involved in cell culturing, MTT assay and statistical analysis. LK coordinated field work and plant sourcing. JNE was involved in coordination and helped to revise the manuscript. SDV assisted with the concept and design of the study and provided technical advice. Authors read and approved the final manuscript.

## Pre-publication history

The pre-publication history for this paper can be accessed here:

http://www.biomedcentral.com/1472-6882/11/14/prepub
